# Color Enhancement in Endoscopic Images Using Adaptive Sigmoid Function and Space Variant Color Reproduction

**DOI:** 10.1155/2015/607407

**Published:** 2015-05-18

**Authors:** Mohammad S. Imtiaz, Khan A. Wahid

**Affiliations:** Department of Electrical and Computer Engineering, University of Saskatchewan, Saskatoon, SK, Canada S7N 5A9

## Abstract

Modern endoscopes play an important role in diagnosing various gastrointestinal (GI) tract related diseases. The improved visual quality of endoscopic images can provide better diagnosis. This paper presents an efficient color image enhancement method for endoscopic images. It is achieved in two stages: image enhancement at gray level followed by space variant chrominance mapping color reproduction. Image enhancement is achieved by performing adaptive sigmoid function and uniform distribution of sigmoid pixels. Secondly, a space variant chrominance mapping color reproduction is used to generate new chrominance components. The proposed method is used on low contrast color white light images (WLI) to enhance and highlight the vascular and mucosa structures of the GI tract. The method is also used to colorize grayscale narrow band images (NBI) and video frames. The focus value and color enhancement factor show that the enhancement level in the processed image is greatly increased compared to the original endoscopic image. The overall contrast level of the processed image is higher than the original image. The color similarity test has proved that the proposed method does not add any additional color which is not present in the original image. The algorithm has low complexity with an execution speed faster than other related methods.

## 1. Introduction

Visual quality of color images plays an important role in medical image diagnosis. Wireless capsule endoscopy (WCE) is an established methodology that offers medical doctors the capability of examining the interior of the small intestine with a noninvasive procedure [1]. However, due to power and hardware limitations, the image quality in WCE is lower than high definition wired endoscopy [2]. Some GI tract related diseases, such as stomach and colon cancers and ulcerative colitis, are now of great threats to human's health [1]. Different such GI diseases can be prevented and cured by means of early detection. Despite several benefits of WCE, the images acquired by this technique are often not clear enough to see the mucosa structure, tissue and vascular characteristics of the digestive tract compared with traditional endoscope, which effects the detection accuracy and increase the miss rate during clinical diagnosis [1, 3–5]. This is why new techniques are being constantly persuaded to enhance certain mucosal or vascular characteristics so that abnormal growths can be visualized better.

There are both in-chip and postprocessing systems that can enhance certain mucosal or vascular characteristics. Among the in-chip technologies, narrow band imaging (NBI) [6] and autoflorescence imaging (AFI) [7] are worth mentioning. There are two types of NBI systems: one is the RGB sequential illumination system, where narrow spectra of red, green, and blue lights centered on 415 nm, 445 nm, and 500 nm, respectively, are used for tissue illumination [8]. In another type of NBI system, a band-pass filter with bandwidths of 30 nm and central wavelengths of 415 nm (for blue) and 540 nm (for green) is used to generate NBI images [6]. On the other hand, in AFI system, a special rotating color filter wheel is used in front of the xenon light source to sequentially generate blue light (390–470 nm) and green light (540–560 nm) for tissue illumination [7]. All of these techniques eventually increase the hardware complexity and power consumption of the endoscopic system. Virtual chromoendoscopy (CE) in contrast is a postprocessing system that decomposes images into various wavelengths and produces reconstructed image with enhanced mucosal surface [9]. Several researchers concluded that NBI appears to be a less time-consuming and equally effective alternative to CE for the detection of neoplasia, but with higher miss rate [3]. Additionally, neither NBI nor CE can improve the adenoma detection or reduce miss rates during screening colonoscopy. No difference has been observed in diagnostic efficacy between these two types of systems [4, 10].

There are some other global and adaptive techniques to enhance contrast and texture information of an image that is, adaptive histogram equalization (AHE) [11], contrast-limited adaptive histogram equalization (CLAHE) [12], high boost filtering (HBF) [13], brightness preserving dynamic fuzzy histogram equalization (BPDFHE) [14]. AHE applies locally varying grayscale transformation to each small blocks of the image, thus requiring the determination of the block size [15]. CLAHE operates on small regions in the image, often called tiles, instead of the entire image, based on user assigned parameters. Finally, the neighboring tiles are combined using bilinear interpolation to eliminate artificial included boundaries.

Two drawbacks of this technique are noise enhancement in smooth regions and image dependency of the contrast gain limit [15]. HBF emphasizes high frequency components without eliminating the low frequency. It may add distortions in the smoothing regions due to over filtering. BPDFHE is the modification of the brightness preserving dynamic histogram equalization (BPDHE) [16] that preserves the brightness and improves contrast enhancement abilities while reducing its computational complexity. However, it introduces additional artifacts depending on the variation of gray level distribution [17] which may lead to inaccurate diagnosis.

In this paper, a versatile endoscopic image enhancement and color reproduction method is proposed which can improve the detection rate of anomalies present in GI images. The image enhancement is achieved in two stages: image enhancement at gray level followed by space variant chrominance mapping color reproduction. Image enhancement is achieved in two steps using adaptive sigmoid function and uniform distribution of sigmoid pixels. This is somewhat similar to our previous work [18], where the enhancement is achieved by applying histogram equalization followed by adaptive sigmoid function; this can however enhance the desired mucosa and vascular features but cannot preserve the brightness of the image. As a result, in this work modified adaptive sigmoid function using precalculated gain and cutoff value is applied first to preserve the brightness of the gray image. The contrast level is enhanced in the next stage using histogram equalization.

Secondly, space variant color reproduction is achieved by generating a real color map by transferring and modifying old chrominance values either from theme image or input image. The proposed method can be useful in the following scenarios.

- In white light imaging (WLI), white light is used for illuminating the GI tract and color images are generated by the endoscope. Using the proposed method, any low-contrast color WLI image can be enhanced at grayscale level and then be colorized with its original color, which can help the gastroenterologists to better inspect the vascular and mucosa structures.
- It can be used in colorizing a grayscale image using the tone of a different color theme image. This is useful when only grayscale image is available (the corresponding color image is either not available or distorted). Secondly, it is useful in saving power and bandwidth during transmission in wireless capsule endoscopy (WCE). Instead of transmitting all color images from the electronic capsule, it can only transmit one color image followed by 3 or 4 grayscale images. Using the proposed method, these grayscale images can be later colorized using the first color image as the theme image.
- In narrow band imaging (NBI), lights of 415 nm and 540 nm wavelengths are used to illuminate the mucosa surface; the reflected light from the mucosa is captured in a monochromic CCD image sensor [19]. The grayscale images from the CCD image sensor are then passed to an image processor where a pseudocolor is added to the images [20]. Using the proposed method, the grayscale NBI images can be further enhanced for better visibility of the mucosa structure; pseudocolors can then be added using the tone of any color theme image.

## 2. Proposed Method

**Figure 1:**
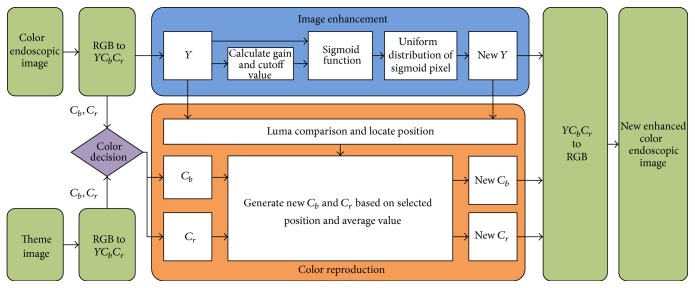
Proposed color image enhancement method.

The proposed method consists of two stages: image enhancement and space-variant chrominance mapping based color reproduction. The method is shown in Figure 1. The stages are briefly discussed below.

### 2.1. Image Enhancement

At first, the color endoscopic image is converted into *YC*_*b*_ *C*_*r*_ color space using (1). Here, *Y* is luminance or luma and *C*_*b*_ and *C*_*r*_ are chrominance components. The color space conversion allows us to process different luma pixels to enhance vascular features and chrominance pixels for color reproduction. Consider

(1) $$\begin{array}{c} \left[\begin{array}{c} Y\\ C_{b}\\ C_{r} \end{array}\right]=\left[\begin{array}{ccc} 0.257 & 0.504 & 0.098\\ -0.148 & -0.291 & 0.439\\ 0.439 & -0.368 & -0.071 \end{array}\right]\left[\begin{array}{c} \text{R}\\ \text{G}\\ \text{B} \end{array}\right]+\left[\begin{array}{c} 16\\ 128\\ 128 \end{array}\right]. \end{array}$$

Here, *Y* is considered grayscale image. After conversion, the proposed method normalizes grayscale image and each chrominance plane between 0 and 1 using (2). Consider

(2) $$\begin{array}{c} N_{\mathrm{norm}}\left(x\right)=\frac{x-x_{\min}}{x_{\max}}. \end{array}$$

Here, *x*_min⁡_ and *x*_max⁡_ are minimum and maximum pixel values. Later, the normalized grayscale image is enhanced using adaptive sigmoid function and uniform distribution.

#### 2.1.1. Adaptive Sigmoid Function

The proposed method uses contrast manipulation techniques for image enhancement. Generally, contrast manipulation technique can be performed either globally or adaptively. Global techniques apply a transformation to all image pixels, while adaptive techniques use an input-output transformation that varies adaptively with local image characteristics. Our method transforms the pixel values adaptively using sigmoid function.

In general, a sigmoid function is real valued and differentiable, having either a nonnegative or nonpositive first derivative that is bell shaped. It has been used in several researches related to image processing [25–27]. Using *x* for the input, the sigmoid function is given below:

(3) $$\begin{array}{c} f\left(x\right)=\frac{1}{\left(1+e^{-x}\right)}. \end{array}$$

In the training mode, we have observed that in a certain exponent the image highlights some vascular characteristics and mucosa structure, which are not clearly visible in the original image. To control the exponent, we have introduced two coefficients in the sigmoid function. Using *x* for the input, *g* for gain, and *k* for cutoff, the modified sigmoid function is expressed below:

(4) $$\begin{array}{c} f\left(x\right)=\frac{1}{\left(1+e^{g(k-x)}\right)}. \end{array}$$

**Figure 2:**
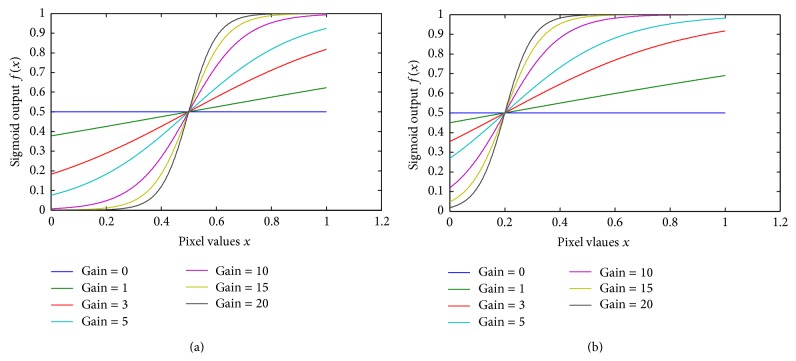
Sigmoid effect on pixel for different gain values (a) with 0.5 cutoff; (b) with 0.2 cutoff.

The cutoff value determines the midpoint of the input curve and the gain controls the amount of bending. These two parameters give us the control to train the proposed method to generate a certain exponent that highlights some vascular characteristics. Let, *x* = 0,0.1,0.2,…, 1 normalized image pixel values where sigmoid function (4) is applied.  Figure 2 presents the sigmoid curve of input pixel values based on different cutoff and gain.

**Figure 3:**
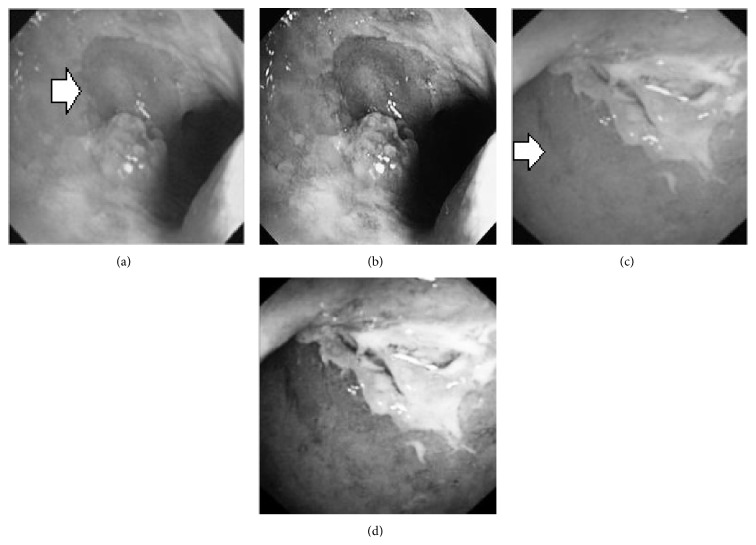
(a) Original image with defected polyp and (c) Crohn's disease; (b) and (d) adaptive sigmoid images of (a) and (c), respectively.

These parameters (gain and cutoff) can control the overall brightness and contrast level of the image too. The cutoff value controls the amount of brightness and the gain controls the consecutive difference between pixels. To maintain the exponent into desired level, we have proposed algorithms to generate cutoff and gain value. Based on the input pixel values, (5) generate specific cutoff and gain value. Later on, these values are used in (4) to generate the sigmoid image. Consider

(5) $$\begin{array}{c} k=\frac{\sum_{i=1}^{n}x_{i}}{n},\\ g=A\times \log\left(\frac{S_{m}}{S_{n}}\right)\times \frac{\sum_{i=1}^{n}x_{i}}{n}, \end{array}$$

where *A* = 100, *S*_*m*_ = 6, *S*_*n*_ = 5, *x*_*i*_ is the pixel values of *i*th position and *n* is the number of pixel. These values are heuristically collected from simulation. First of all, we processed endoscopic images in different combination of gain and cutoff values. The images are collected from Gastrolab [28] and Atlas [29] database and have comments from gastroenterologist; as a result, they can be sub-divided into different disease categories.  Figure 3 shows some examples of the original and corresponding sigmoid images. The abnormalities in the images may be identified, but not the tissue and vascular characterization (as marked with an arrow in Figures 3(a) and 3(c)). It is noted that mucosa structure, tissue and vascular characteristics are important since by analyzing them the status of gastric glands and pits can be investigated [30–32].

During simulation, we observed that in certain cases, with gain in a range of 7.5–8.5 and cutoff in a range of 0.4–0.5, the tissue and vascular characterization are highly visible. To keep the gain and cutoff in that desired range, we propose (5). For better illustration, we have presented sigmoid images processed with different combination of gain and cutoff values in Table 1. Here, the effects on images for different combination of gain and cutoff values are observed. For example, Image #1 and #5 have low intensity; image #2 has high brightness; image #3 and #4 have highlighted tissue and vascular characterization.

#### 2.1.2. Uniform Distribution of Sigmoid Pixels

In the next stage, the sigmoid pixels are uniformly distributed to increase the contrast level. It helps to visualize the vascular characteristic of darker part of an adaptive sigmoid image. It is employed by effectively spreading out the most frequent intensities.

Let, *f* be a given sigmoid image represented as *i* by *j* matrix of integer pixel intensities ranging from 0 to 255. Let, *p* denotes the normalized histogram of *f* with bin for possible intensities. So,

(6) $$\begin{array}{c} p_{m}=\frac{\text{Number}\;\text{of}\;\text{pixels}\;\text{with}\;\text{intensity}\;m}{\text{total}\;\text{number}\;\text{of}\;\text{pixels}}, \end{array}$$

where *m* = 0,1, 2,…, 255. The uniformly distributed sigmoid image Ψ is defined as,

(7) $$\begin{array}{c} \Psi_{i,j}=\text{floor}\left(\left(L-1\right)\overset{f_{i,j}}{\underset{n=0}{\sum }}p_{m}\right), \end{array}$$

where floor() maps to the largest integer but lesser than the number. Normally, the cumulative distribution function (CDF) of an image does not form a horizontal line, that means, the pixel values are not equally likely to occur. In the proposed method, a uniform distribution of sigmoid pixels is achieved by applying (6) and (7); this technique is similar to global histogram equalization.  Table 2 shows the visual comparison of uniform distribution of sigmoid pixels. This uniformly distributed sigmoid image Ψ_*i*,*j*_ is later treated as new enhanced grayscale image (Ψ).

### 2.2. Color Reproduction

In the second stage of the proposed method, we apply color reproduction. It is a computer-assisted process of adding color to a monochrome image [33, 34]. In the proposed method, it is possible to retrieve the original color with a better tone or add pseudocolor using a theme image. This choice is controlled by the user through the “color decision” module (see Figure 1) which selects the chrominance components.

**Case 1 .**

To retrieve original color, we first create new *C*_*b*_ and *C*_*r*_ planes by matching the original *C*_*b*_ and *C*_*r*_ values for corresponding *Y* pixels from the original grayscale image. First of all, the positions of all *C*_*b*_ and *C*_*r*_ values in the plane for a particular *Y* pixel are identified as expressed by (8):

(8) $$\begin{array}{c} \left[m,n\right]=\text{locate}\left(Y^{}-Y_{i,j}^{}\right). \end{array}$$

Here, *Y* is normalized grayscale image, *Y*_*i*,*j*_ is a pixel of normalized grayscale image and [*m*, *n*] holds one or multiple positions. These positions will allow us to generate new chrominance planes. Two scenarios may occur: (a) if only one chrominance value is found, it places that value in the corresponding positions in the new *C*_*b*_ and *C*_*r*_ planes. (b) Otherwise, if multiple chrominance values are found, it generates a new chrominance value using (9) and places it in the corresponding positions of the new *C*_*b*_ and *C*_*r*_ planes

(9) $$\begin{array}{c} \overset{-}{x}=\frac{\sum_{i=1}^{n}x_{i}}{n}. \end{array}$$

These steps continue until all pixels of the grayscale image are scanned. The new *C*_*b*_ and *C*_*r*_ will have the same dimension of the original grayscale image. Later, the enhanced grayscale image (Ψ) and the new *C*_*b*_ and *C*_*r*_ images are converted back to RGB image using (10)

(10) $$\begin{array}{c} \left[\begin{array}{c} \text{R}\\ \text{G}\\ \text{B} \end{array}\right]=\left[\begin{array}{ccc} 1.164 & 0 & 1.596\\ 1.164 & -0.392 & -0.813\\ 1.164 & 2.017 & 0 \end{array}\right]\left[\begin{array}{c} \Psi -16\\ C_{b}^{\text{new}}-128\\ C_{r}^{\text{new}}-128 \end{array}\right]. \end{array}$$

**Case 2 .**

To add pseudocolor, a theme image is required. It is applicable when only grayscale image or no color information is available. As the color information in an endoscopic image dictates clinical decision, the selection of theme image is very important. The theme image must be selected from the nearby location or region of GI tract. After selecting a proper theme color image, it is converted into *YC*_*b*_ *C*_*r*_ space. Then, we create new *C*_*b*_ and *C*_*r*_ planes by matching the chrominance values of the theme image for the corresponding enhanced pixel (Ψ_*i*,*j*_). Now, similar procedure as given in (8) is followed to find the new *C*_*b*_ and *C*_*r*_ planes (given in (11))

(11) $$\begin{array}{c} \left[m,n\right]=\text{locate}\left(Y_{t}-\Psi_{i,j}\right). \end{array}$$

Here, *Y*_*t*_ is normalized theme grayscale image, Ψ_*i*,*j*_ is a pixel of enhanced grayscale image and [*m*, *n*] holds one or multiple locations. These locations allow us to generate the new chrominance plane with respect to the enhanced and theme grayscale images. Here, the chrominance values are generated from the *C*_*b*_ and *C*_*r*_ planes of the theme image.

Three scenarios may occur: (a) if only one chrominance value is found, it places that value in the corresponding positions in the new *C*_*b*_ and *C*_*r*_ planes. (b) if multiple chrominance values are found, it generates a new chrominance value using (9) and places it in the corresponding positions of the new *C*_*b*_ and *C*_*r*_ planes (c) if no chrominance value is found, it reads the chrominance value respect to the positions of Ψ_*i*,*j*_ in theme *C*_*b*_ and *C*_*r*_ planes and places it in the corresponding position of the new *C*_*b*_ and *C*_*r*_ planes. These steps continue until all pixels of the enhanced grayscale image are scanned. The new *C*_*b*_ and *C*_*r*_ will have the same dimension of the original grayscale image. Later, the enhanced grayscale image (Ψ) and the new *C*_*b*_ and *C*_*r*_ images are converted back to RGB image using (10).

**Figure 4:**
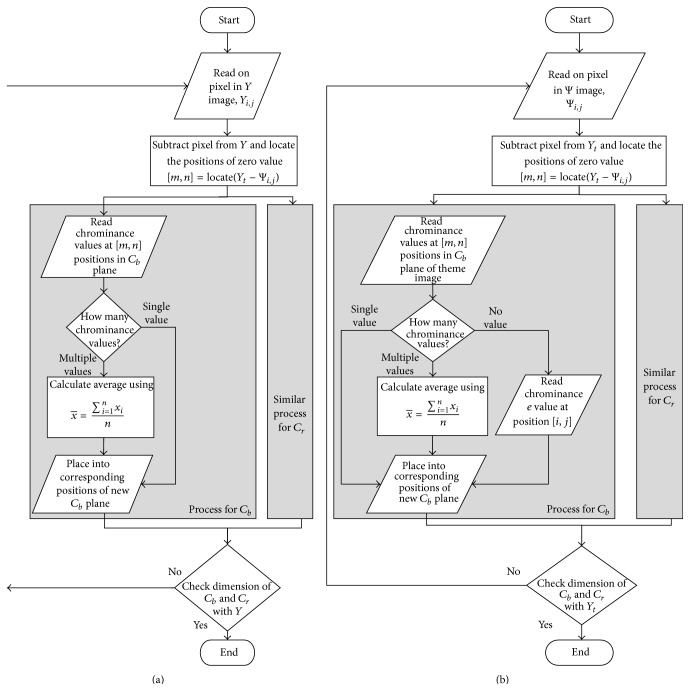
Flow chart: (a) Case 1: to retrieve original color; (b) Case 2: to add pseudocolor from theme image.

**Figure 5:**
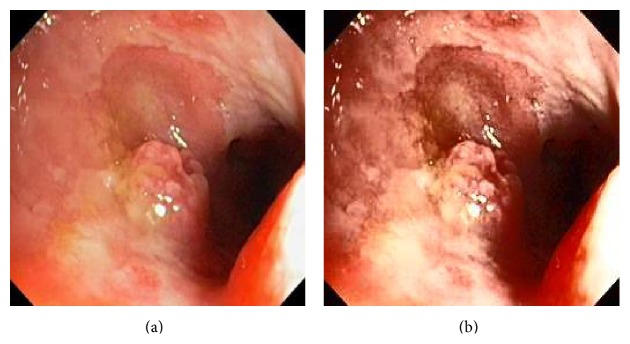
(a) Original image and (b) enhanced color image (color reproduced from original image).

**Figure 6:**
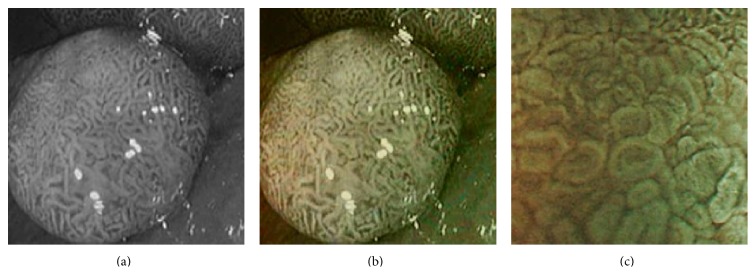
(a) Original grayscale image (no color information is available) (b) enhanced color image (color reproduced from theme image shown in (c)).

In Figure 4, the flow chart of the color reproduction algorithm is presented. Some reconstructed images for the two cases are shown in Figures 5 and 6. It can be seen that the proposed method enhances color information in all reconstructed images.

## 3. Results and Discussion

In order to evaluate the performance of the proposed algorithm, we have applied it to several endoscopic images collected from Gastrolab [28] and Atlas [29]. The results are summarized below in four categories.

### 3.1. Category 1: Low-Contrast Color Images

In this case, the input image is first enhanced on gray level and then color added. The chrominance values of the original input image are used for color reproduction. As a result, the output image has similar color tone with enhanced features as shown in Table 3. It can be seen from the table that the vascular and other mucosa structures are better visible and highlighted in the output images, which can help the gastroenterologists in better diagnosis.

### 3.2. Category 2: Low-Contrast Grayscale Images

In this case, we show examples where low-contrast grayscale images are used (i.e., color information is not available for these images). The grayscale images are first enhanced and then colorized using a theme image. The choice of the theme image is important as it may add color distortion if not properly chosen. As a result, we choose a theme image from the same or similar physical location of the GI tract. The results of the enhanced color images are shown in Table 4 along with the corresponding theme images.

### 3.3. Category 3: Raw NBI Images

In the next experiment, we applied our algorithm on several NBI images (grayscale in nature) as shown in Table 5. The raw NBI images are enhanced first and then a color theme image is used to generate pseudocolor. The theme images are chosen the same way as described before. We can see from the table that the output images have much better visibility of the mucosa structure compared to the grayscale images.

### 3.4. Category 4: Image Transmission in WCE

The proposed color generation method is very useful in saving power consumption during transmission in wireless capsule endoscopy (WCE). Instead of transmitting all color images from the electronic capsule (which takes 24 bits per pixel per image), it can only transmit one color image at the beginning followed by a defined number of grayscale images (8 bits per pixel per image). Using the proposed method, these grayscale images will be later colorized using the first color image at the receiver. In Table 5, we show the results of such case where the R, G and B components of frame 1 are transmitted first. Then only the luminance (*Y*) components of frame 2, 3, 4, and 5 are transmitted. At the receiver, these frames 2–5 are reconstructed using the proposed color reproduction method taking frame 1 as the theme image. Later on, the color reconstructed images are compared with the original color video sequences. In conventional case, the R, G, and B components of all frames are transmitted. For the given case, for five frames, it will require a total of 120 bits per pixel (i.e., 24 × 5). On the other hand, using the proposed method, it will require only 56 bits per pixel (i.e., 24 + 8 + 8 + 8 + 8) which results in a saving of 53% during the transmission. More saving will be achieved using the number of grayscale frames is increased. The original color video frames are also shown in Table 6 for comparison. Here we see that the reconstructed output images have the same color as compared with the original color video frames with a power saving of 53%.

It should be noted here that, the previous work [18] was only applied to color images whereas the proposed method can be applied to both color and grayscale images. As a result, low-contrast gray (category 2) and NBI raw (category 3) images can be colorized using the method using a theme image. This feature also makes the algorithm helpful in saving power during WCE image transmission (category 4).

## 4. Performance Analysis

In the following section, the performance of the proposed scheme is evaluated using focus value, statistic of visual representation, measurement of uniform distribution, color similarity test, color enhancement factor (CEF) and time complexity. The results are discussed below.

### 4.1. Focus Value

In our method, image enhancement is achieved by adaptive sigmoid function and uniform distribution of sigmoid pixels. As a result, the overall information of sharp counters and contrast is increased. These changes of an image are evaluated using focus value [35]. Focus value is a mathematical representation of the ratio of AC and DC energy values of a Discrete Cosine Transform (DCT) of an image [36]. Let *E*_AC⁡_ be the AC values and *E*_DC_ the DC value of a DCT image. *E*_AC⁡_ values carry the information related to high frequency component (i.e., changes of contrast level, sharp counters and crisp edges) of an image. On the other hand, *E*_DC_ value carries only the information related to low frequency components (i.e., luminance or brightness). The expressions are given below:

(12) $$\begin{array}{c} E_{\text{DC}}={\left(F_{\text{DC}}\left(u,v\right)\right)}^{2},\\ E_{\mathrm{AC}}=\overset{n}{\underset{u=1}{\sum }}\;\overset{m}{\underset{v=1}{\sum }}{\left(F_{\mathrm{AC}}\left(u,v\right)-\bar{F_{\mathrm{AC}}\left(u,v\right)}\right)}^{2}. \end{array}$$

Here, *u* and *v* represent the row and column of the DCT image, *F*_DC_ is the DC part and *F*_AC⁡_ is the AC part of DCT image. The resultant of the ratio of *E*_AC⁡_ and *E*_DC_ is the focus value *F*_*S*_ as given by

(13) $$\begin{array}{c} F_{S}=\frac{E_{\mathrm{AC}}}{E_{\text{DC}}}. \end{array}$$

If the overall information of sharp counters, crisp edges and contrast of enhanced image is higher than the original image, then *F*_*S*_ of the enhanced image will be higher than that of the original image and vice versa. We have compared our method in terms of focus value using 60 sample images with other methods like AHE [11], CLAHE [12], HBF [13] and BPDFHE [14]. The results are presented in Table 7. Here, we see that the focus values of the proposed method are relatively higher compared to the other methods.

### 4.2. Statistic of Visual Representation

Next, we used statistic of visual representation [37] to measure the contrast and intensity distortion between two images. Equations (14) represent statistic visual representation. Consider

(14) $$\begin{array}{c} C=\frac{\sigma_{\text{out}}-\sigma_{\text{in}}}{\sigma_{\text{in}}},\\ L=\frac{L_{\text{out}}-L_{\text{in}}}{L_{\text{in}}}, \end{array}$$

where *σ*_out_ and *L*_out_ are the variance and mean of enhanced image; *σ*_in_ and *L*_in_ are the variance and mean of original image, respectively. Here, *C* defines the percentage of increment or decrement of contrast level and *L* defines the percentage of increment or decrement in intensity level. In our experiment, we used 60 grayscale images. The results are presented in Table 8. We can see that the *C* and *L* of the first image using proposed method are 1.0636 and 0.0716, which means that the contrast and intensity level of proposed image are 103.6 and 7.16 times higher than the original image, respectively. Here, the negative sign denotes the decrement. It is noticeable that the proposed method's contrast level and intensity level are higher compare to the other method.

### 4.3. Measurement of Uniform Distribution

Here, we calculate the uniform distribution of R, G, and B planes by calculating entropy [38, 39]. The more the uniform distribution of color planes, the better the color enhancement. The entropy of *n* distributed signals is defined by

(15) Hx1,x2,…,xn =−∑x1 ∑x2⋯∑xnPx1,x2,…,xn×log2 Px1,x2,…,xn.

First, we have showed the advantage of using proposed color reproduction in Table 9. Here, in image (a), we used the proposed image enhancement algorithm on the luminance plane and left the chrominance planes unchanged. In image (b), we applied proposed image enhancement on luminance and color reproduction on the chrominance planes. From both images, it is noticeable that the image in (a) without color reproduction does not preserve brightness and shows imbalance saturation level. On contrary, the image in (b) with color reproduction has much balanced saturation and it preserves the overall brightness. It happens because *YC*_*b*_ *C*_*r*_ is a nonuniform and nonorthogonal color space. That is why we need to manipulate both luminance and chrominance in such a way that the correlation does not break and preserve the brightness along with the color saturation level. Additionally, our method achieves a higher entropy value, which means that it produces a more uniform histogram. The entropy value of image (b) is 7.6237 which is higher than that of image (a) that is 7.4961.

Table 10 shows the performance comparison with other related methods. In shows that the proposed method produces images with enhanced and highlighted mucosa structures. The results are also summarized in Table 11.

### 4.4. Color Similarity Test

To validate the results statistically, the color similarity between the original and color reproduced images is evaluated using several performance metrics such as, CIE94 delta-*E* color difference [40], mean structure similarity index (MSSIM) [41] and structure and hue similarity (SHSIM) [42]. The purpose is to show that our color reproduction method does not add any additional color. CIE94 is used to measure the color differences between processed and original image in LAB color space. In CIE94, Δ*E*_94_^*^ ≈ 2.3 indicates that the color difference between two images is the lowest. MSSIM are used to measure color similarity in the chrominance planes in *YC*_*b*_ *C*_*r*_ color space. SHSIM is used to measure the hue and structure similarity between processed and original image in HSV color space. Here, we have used 60 trial images to evaluate the color similarity index. The results are compared with other color reproduction methods and presented in Table 12. It can be seen that the average MSSIM and SHSIM indices are higher than others in our scheme with a color difference Δ*E*_94_^*^ close to 2.3. All these values indicate that the colorized images are very close to the original images.

### 4.5. Color Enhancement Factor (CEF)

**Figure 7:**
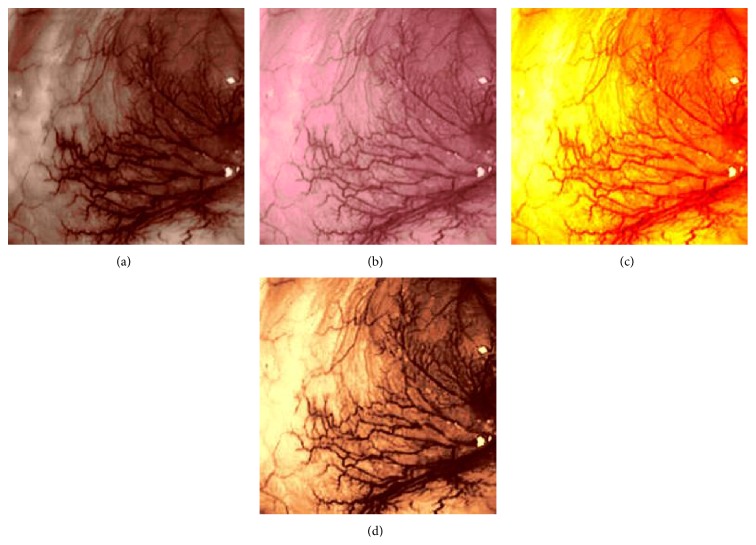
Enhanced color images using different color reproduction algorithms. (a) References [23, 24]. (b) Reference [21]. (c) Reference [22]. (d) Proposed.

We have also evaluated our scheme in terms of color enhancement. Here, we have used a no-reference performance metric called colorfulness matric (CM) [43]. The CM measurement is based on the mean and standard deviations of two axes opponent color representation with, *α* = R − G and *β* = (1/2)(R + G) − B. The metric is defined as

(16) $$\begin{array}{c} \text{CM}=\sqrt{\sigma_{\alpha }^{2}+\sigma_{\beta }^{2}}+0.3\sqrt{\mu_{\alpha }^{2}+\mu_{\beta }^{2}}, \end{array}$$

where *σ*_*α*_ and *σ*_*β*_ are standard deviations of *α* and *β*, respectively. Similarly, *μ*_*α*_ and *μ*_*β*_ are their means. However, in our comparison, we have used the ratio of CMs between the enhanced and original image for observing the color enhancement factor (CEF). If CEF < 1, than the original image is better compared to the enhanced image in terms of color image enhancement. CEF with value 1 indicates that there is no difference between the enhanced and original image in terms of color enhancement. The results have been presented in Tables 13 and 14. Here we can see that CEF values of the proposed method are highest compared to other enhancement methods which indicates that our scheme performs better in terms of color enhancement.  Figure 7 shows some reconstructed images.

### 4.6. Algorithm Complexity

The time required to generate an enhanced color image for different image sizes using the proposed method and other related works [21–24] are shown in Table 15. The experiment was conducted on a PC having Intel (R) Pentium(R) dual CPU @ 2.00 GHz and 6 GB of RAM. Here, it is noticeable that the proposed method is the fastest method including both image enhancement and color reproduction. For an image of *n* pixels, the proposed algorithm has linear computational time complexity, *O*(*n*). The average simulation time of proposed method for 256 × 256 images is approximately 22 seconds and for 512 × 512 images is approximately 85 seconds. The work in [21, 23, 24] have significantly higher execution time when compared with the proposed method. Although the execution time of [22] is lower than ours, the quality of the color reproduction is much worse as shown in Figure 7.

## 5. Conclusion

In this paper, we have presented an image enhancement and color reproduction method for endoscopic images. The work focuses on enhancing the mucosa structures present in endoscopic image. The proposed color image enhancement is achieved in two stages: image enhancement at gray level followed by space variant chrominance mapping color reproduction. Image enhancement is achieved in two steps: adaptive sigmoid function and uniform distribution of sigmoid pixels. Secondly, space variant color reproduction is performed by generating a real color map by transferring and modifying old chrominance values either from theme image or input image. The quality of the generated enhanced colored images is evaluated using several standard performance metrics, which show that the features are highlighted on the new processed images.

## Figures and Tables

**Table 1:** Sigmoid image with different combination of gain (*g*) and cutoff values (*k*).

Number	Original gray scale image	Sigmoid image	Used gain and cutoff values
1	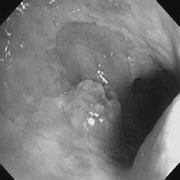	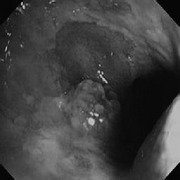	*g* = 5 *k* = 0.7

2	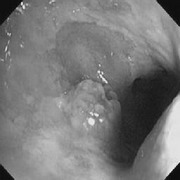	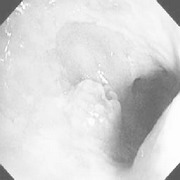	*g* = 6 *k* = 0.1

3	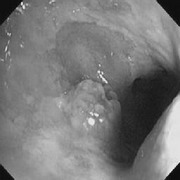	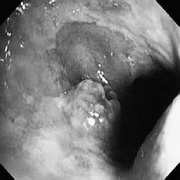	*g* = 8 *k* = 0.5

4	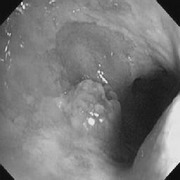	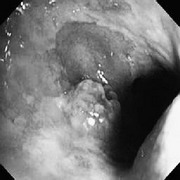	*g* = 7.5 *k* = 0.4

5	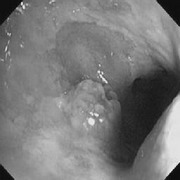	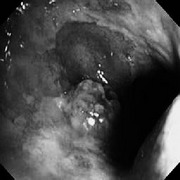	*g* = 9 *k* = 0.6

**Table 2:** The Comparison between processed sigmoid image and uniformly distributed sigmoid image.

Image type	Cumulative distribution function (CDF)	Histogram of image
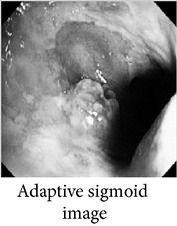	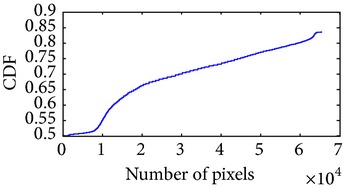	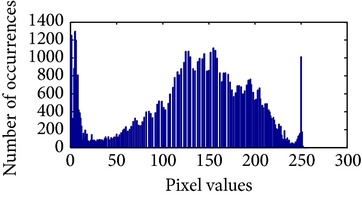

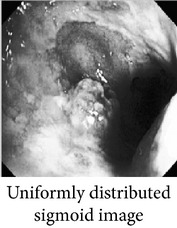	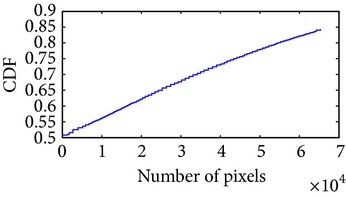	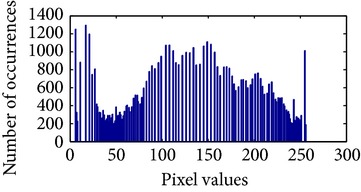

**Table 3:** Category 1: Enhancement of colored WLI images (where input image is used as theme image).

Number	Input original image (color)	Enhanced grayscale image	Output enhanced color image
1	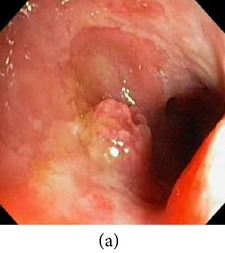	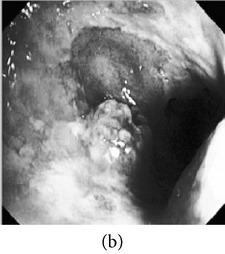	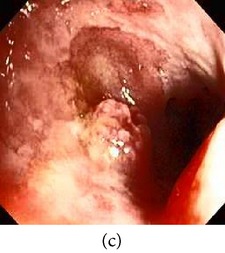

2	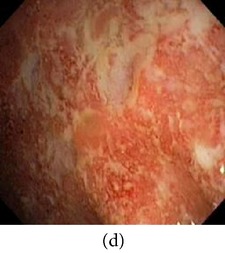	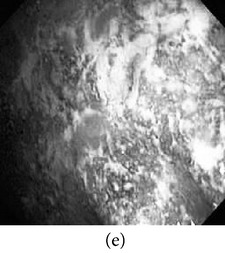	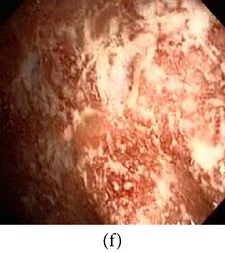

3	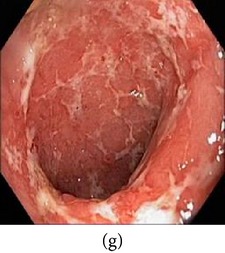	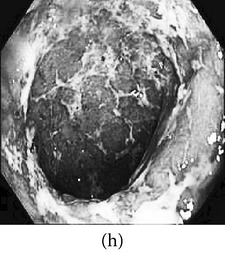	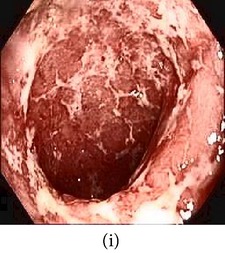

4	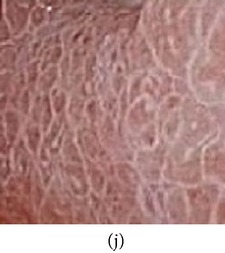	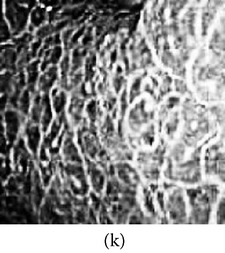	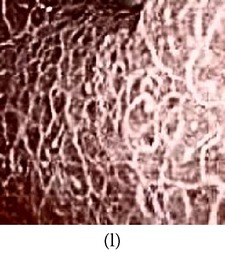

**Table 4:** Category 2: Enhancement of grayscale WLI images (here, no original color image is available, so color image from similar location of the GI tract is used as theme image).

Number	Input original image (grayscale)	Enhanced grayscale image	Theme image	Output enhanced color image
1	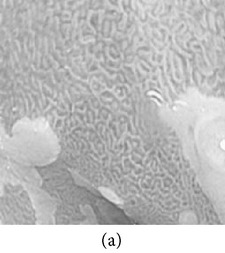	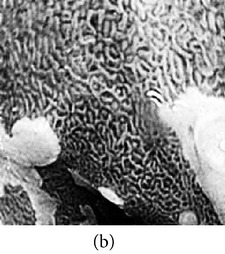	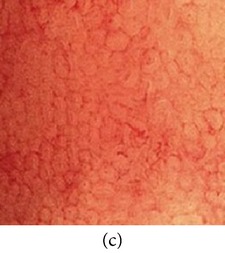	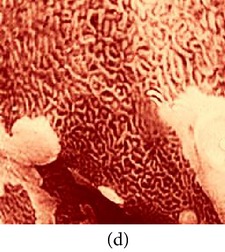

2	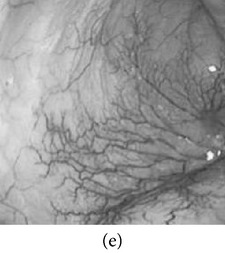	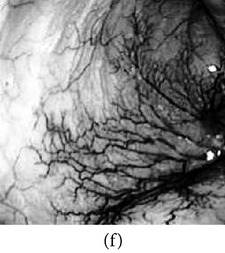	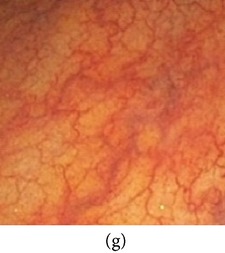	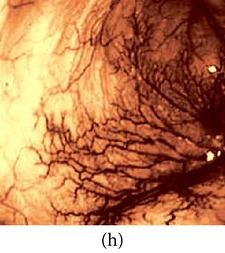

3	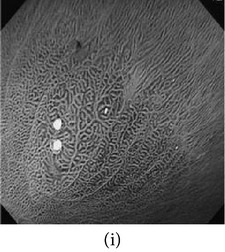	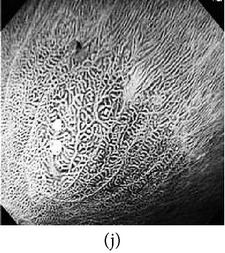	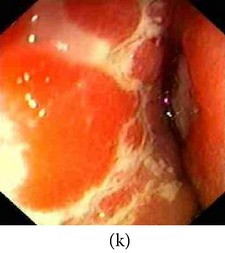	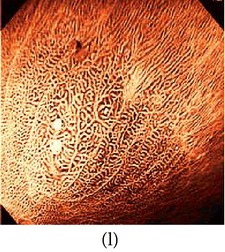

4	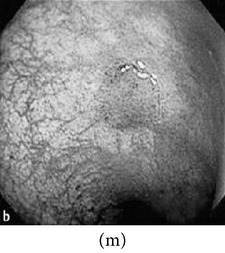	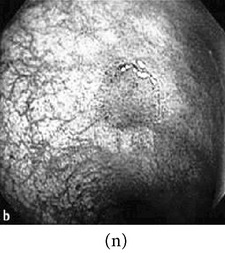	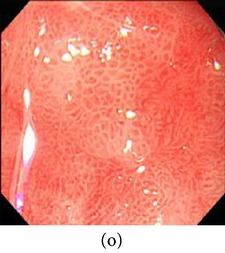	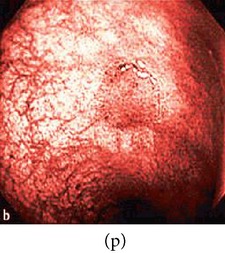

**Table 5:** Category 3: Enhancement and color reproduction of grayscale NBI images.

Number	Input RAW NBI image	Enhanced grayscale image	Theme image	Output enhanced color image

1	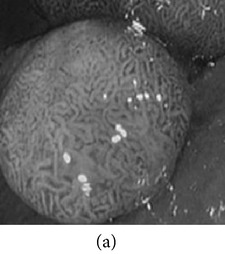	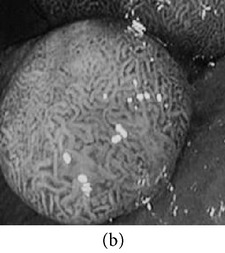	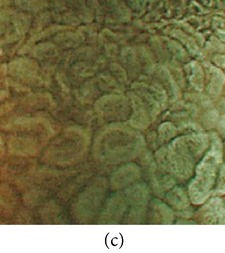	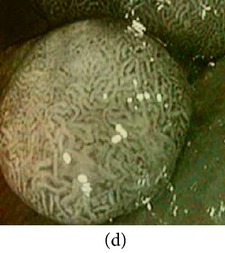

2	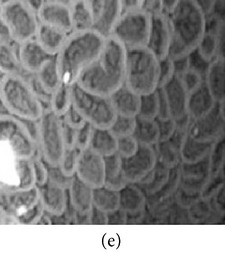	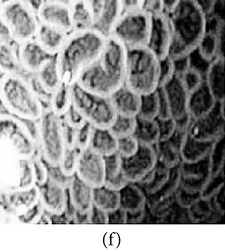	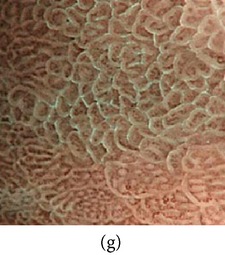	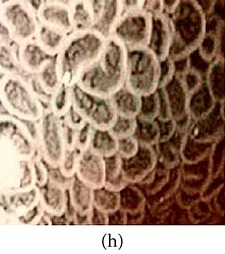

3	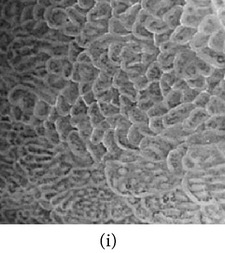	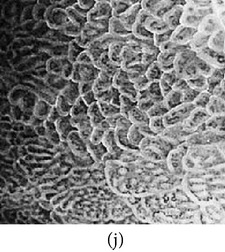	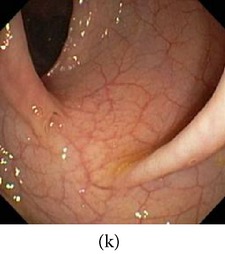	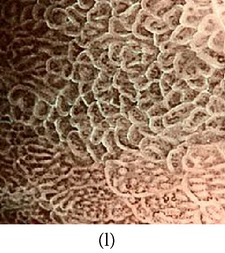

**Table 6:** Category 4: Reproduction of color frames from grayscale frame in WCE video; no enhancement was applied.

Frame number	1	2	3	4	5
Original video frames	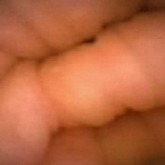	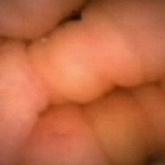	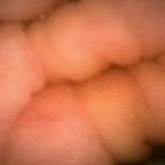	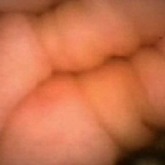	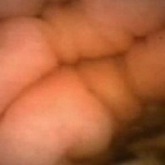

Input images	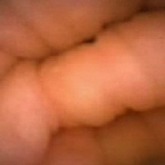	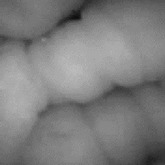	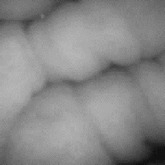	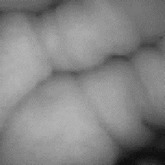	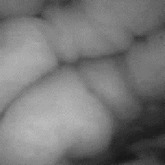

Proposed color images (Frame number 1 used as theme image)	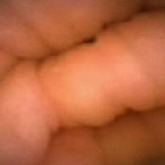	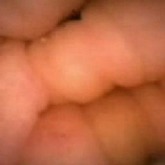	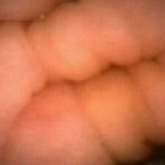	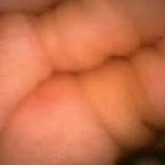	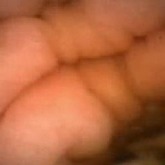

**Table 7:** Comparisons of focus value with other related works.

Image number	Focus value					
	Original	AHE [11]	CLAHE [12]	HBF [13]	BPDFHE [14]	Proposed
1	15.64	17.50	22.96	22.86	22.04	**42.10**
2	12.52	16.75	20.14	19.33	17.11	**42.52**
3	18.01	19.20	23.18	22.40	22.00	**41.57**
4	13.66	18.85	22.01	20.97	20.51	**42.00**

Average of 60 endoscopic images	13.77	19.08	21.11	20.49	19.49	**41.17**

**Table 8:** Comparisons of statistic of visual representation with other related works.

Image number	Contrast measurement					Intensity measurement				

AHE [11]	CLAHE [12]	HBF [13]	BPDFHE [14]	Proposed	AHE [11]	CLAHE [12]	HBF [13]	BPDFHE [14]	Proposed

1	0.3373	0.4735	0.4285	0.2025	1.0636	−0.1781	0.0598	0.0254	0.0065	0.0716
2	0.2767	0.6352	0.2395	0.3608	2.4684	−0.0312	0.1207	0.0095	0.0092	0.2526
3	0.1205	0.2935	0.2395	0.1192	1.12294	−0.1122	0.0410	0.0095	−0.0012	0.1322
4	0.3401	1.1598	0.8235	0.7206	2.0349	−0.1186	−0.0291	0.0134	−0.0100	0.0218

Average of 60 endoscopic images	0.2554	0.6411	0.4149	0.3371	1.6147	−0.1203	0.0381	0.0197	0.0174	0.1748

**Table 9:** Histogram of *R*, *G*, and *B* planes in terms of uniform distribution (a) without and (b) with color reproduction.

Processed image	Histogram of *R* plane	Histogram of *G* plane	Histogram of *B* plane

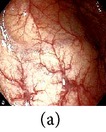	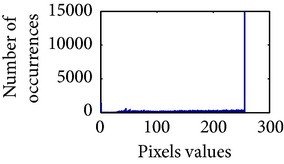	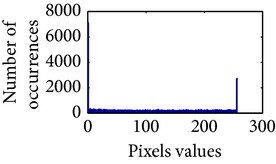	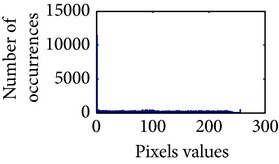

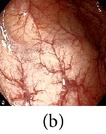	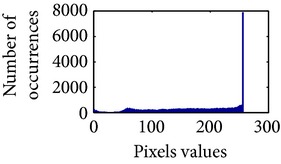	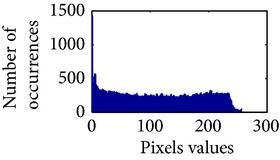	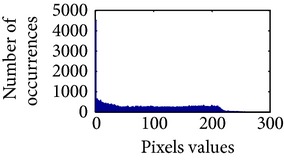

**Table 10:** Comparison of the histogram of *R*, *G*, and *B* planes in terms of uniform distribution.

Processed image	Histogram of *R* plane	Histogram of *G* plane	Histogram of *B* plane
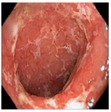 Original	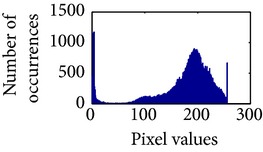	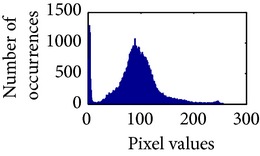	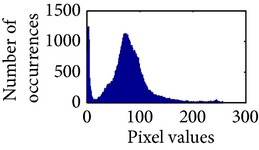

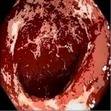 AHE [11]	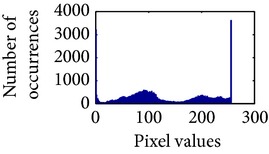	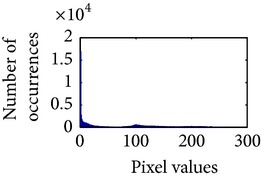	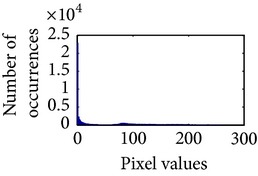

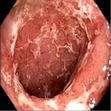 CLAHE [12]	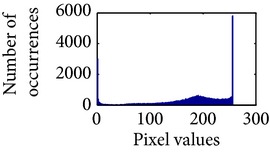	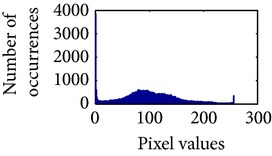	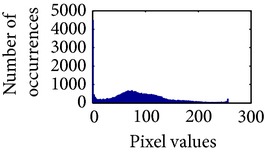

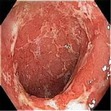 HBF [13]	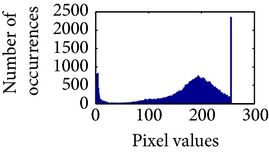	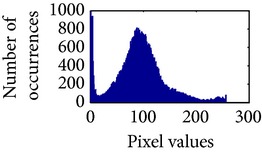	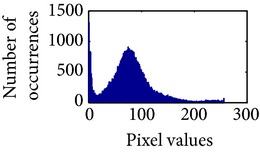

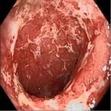 BPDHGE [14]	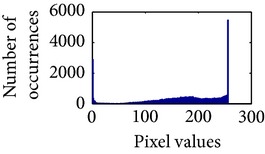	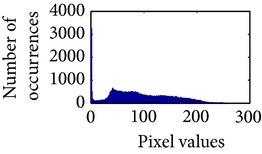	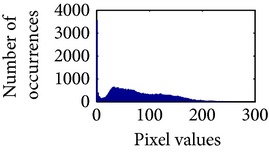

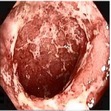 Proposed	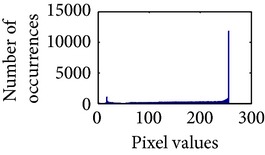	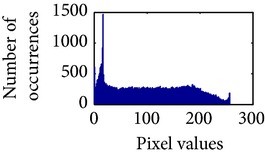	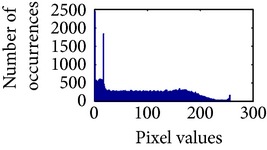

**Table 11:** Comparisons of the measurement of uniform distribution based on entropy with other related works.

Image number	Measurement of entropy					
	Original	AHE [11]	CLAHE [12]	HBF [13]	BPDFHE [14]	Proposed
1	7.25	6.68	7.38	7.33	7.41	**7.46**
2	7.21	6.47	7.48	7.33	7.44	**7.57**
3	7.11	6.38	7.34	7.31	7.38	**7.51**
4	6.52	4.87	7.11	6.77	7.51	**7.70**

Average of 60 endoscopic images	7.41	7.01	7.68	7.31	7.75	**7.91**

**Table 12:** Color similarity assessment.

Similarity between two color images		Δ*E*_94_^*^	MSSIM	SHSIM

Image 1	Image 2

Original image	Proposed	**2.97**	**0.9851**	**0.9992**
	[21]	4.1	0.8605	0.8578
	[22]	0.6	0.8714	0.8001
	[23, 24]	3.01	0.9567	0.9942

**Table 13:** Comparisons of CEF indices with other enhancement works.

Image number	AHE [11]	CLAHE [12]	HBF [13]	BPDFHE [14]	Proposed

1	0.9620	1.1197	1.0355	1.0149	**1.7329**
2	0.9623	1.0989	1.0077	1.0037	**1.8897**
3	0.9446	1.1716	1.0751	1.0547	**1.8812**
4	0.9946	1.2009	1.1913	1.1003	**1.7443**

Average of 60 endoscopic images	0.9661	1.1574	1.0614	1.0411	**1.7477**

**Table 14:** Comparisons of CEF indices with other color reproduction works.

Image number	[23, 24]	[21]	[22]	Proposed
1	1.1059	0.5987	0.5118	**1.7784**
2	1.0991	0.7481	0.5997	**1.6599**
3	1.1007	0.6187	0.6001	**1.8413**
4	1.1972	0.5249	0.4991	**1.7749**

Average of 60 endoscopic images	1.1391	0.5149	0.4977	**1.5621**

**Table 15:** Comparison of simulation speed between proposed method and other related works.

Methodology	Image size	Step 1	Step 2	Total time (sec.)
		Enhancement time (sec.)	Color reproduction time (sec.)	
Proposed	**256 × 256**	**1.909**	**20.614**	**22.523**
	**512 × 256**	**2.597**	**40.119**	**42.716**
	**512 × 512**	**3.182**	**81.779**	**84.961**

[23, 24]	256 × 256	0.39	28.255	28.645
	512 × 256	1.519	72.956	74.475
	512 × 512	1.525	113.31	114.835

[21]	256 × 256	—	64.461	64.461
	512 × 256	—	117.921	117.921
	512 × 512	—	232.146	235.149

[22]	256 × 256	—	0.19	0.19
	512 × 256	—	0.81	0.81
	512 × 512	—	0.47	0.47
